# Women survive longer than men undergoing cytoreductive surgery and HIPEC for appendiceal cancer

**DOI:** 10.1371/journal.pone.0250726

**Published:** 2021-04-30

**Authors:** Noah S. Rozich, Samara E. Lewis, Sixia Chen, Kenneth E. Stewart, Michael B. Stout, William C. Dooley, Laura E. Fischer, Katherine T. Morris

**Affiliations:** 1 Department of Surgery, University of Oklahoma Health Sciences Center, Oklahoma City, OK, United States of America; 2 Department of Biostatistics and Epidemiology, University of Oklahoma Health Sciences Center, Oklahoma City, OK, United States of America; 3 Department of Nutritional Sciences, University of Oklahoma Health Sciences Center, Oklahoma City, OK, United States of America; Chang Gung Memorial Hospital and Chang Gung University, Taoyuan, TAIWAN

## Abstract

**Background:**

We hypothesize that women undergoing cytoreductive surgery (CRS) and hyperthermic intraperitoneal chemotherapy (HIPEC) for peritoneal carcinomatosis from appendiceal cancer will have a survival advantage compared to men.

**Methods:**

The National Cancer Database (NCDB) public user file (2004–2014) was used to select patients with PC undergoing CRS and HIPEC from appendiceal cancer. Univariate and multivariable analyses were performed.

**Results:**

1,190 patients with PC from appendiceal cancer underwent HIPEC and CRS. OS was significantly longer for women than for men, with mean and median OS being 73.8 months and 98.2 months for women vs 58.7 months and 82.5 months for men, respectively (p = 0.0032). On multivariable analysis, male sex (HR: 1.444, 95% CI: 1.141–1.827, p = 0.0022) and increasing age (HR: 1.017, 95% CI: 1.006–1.027, p = 0.0017) were both found to be independent risk factors for worse OS.

**Conclusion:**

Women undergoing CRS and HIPEC for PC from appendiceal origin live longer than men undergoing the same treatment. Increasing age was also found to be independent risk factors for worse survival

## Introduction

Appendiceal mucinous neoplasms (AMN) are a heterogenous and relatively rare group of gastrointestinal (GI) malignancies arising from the appendix, accounting for 0.4%-1.0% of all GI malignancies in the United States [1]. They include a range of histologic subtypes, and compared to cancers of colorectal origin, they tend to behave less aggressively and rarely metastasize outside of the peritoneal cavity. Approximately 53% present with peritoneal metastasis in the form of peritoneal carcinomatosis (PC). Initial presentation can therefore include increased abdominal girth, due to the accumulation of mucinous ascites and peritoneal studding, clinically known as pseudomyxoma peritonei (PMP) [2]. Patients with early stage disease often present with symptoms consistent with acute appendicitis, with up to 32% of patients with AMN diagnosed with acute appendicitis preoperatively [3]. Advanced-staged presentation is more common, and recent studies show a trend of increasing overall incidence and decreasing age at diagnosis [1, 3, 4].

Treatment of local and regional disease typically consists of surgical resection with appendectomy and right-hemicolectomy, respectively, with adjuvant chemotherapy consisting of 5-fluorouracil-based regimens generally reserved for patients with high-risk features [5–7]. Surgical debulking with cytoreductive surgery (CRS) combined with hyperthermic intraperitoneal chemotherapy (HIPEC) has emerged as standard treatment for advanced stage disease with peritoneal involvement [8], with fluorouracil-based neoadjuvant chemotherapy regimens primarily used in patients with high-grade histology [1]. Multiple factors have been evaluated for their impact on outcomes following CRS and HIPEC, including lymph node (LN) metastasis, peritoneal cancer index (PCI) score, histopathology, and the extent and completeness of CRS [1]. While completeness of CRS, favorable histopathology, and lower PCI have been demonstrated to be associated with improved overall survival (OS), positive LN metastasis, incomplete cytoreduction, elevated tumor markers (CEA, CA125, CA19-9), and high-grade tumors have all been demonstrated to be associated with worse OS [9–12].

The impact of sex on outcomes for patients presenting with AMN with PC undergoing CRS and HIPEC has not been extensively explored. While several studies report no difference in survival between sexes [13–15], others note a survival advantage for women compared to men [10, 16, 17]. Previous studies are predominantly single-institution, retrospective analyses, that focus primarily on other prognostic factors and do not explore patient sex as a variable of interest. However, the potential for a survival disparity between sexes with appendiceal cancer is not altogether surprising considering the differences in biology, incidence, and treatment outcomes between men and women for various other cancers, including cancers of colorectal origin. For example, women are more likely to have right-sided colorectal tumors, a lower overall incidence of colorectal cancer (CRC), higher rates of micro-satellite instability in colorectal tumors, and increased overall survival after resection for locoregional disease [18–20]. Furthermore, in patients with PMP of appendiceal origin, studies have demonstrated differences in expression of common tumor suppressor genes and tumor-associated stromal proteins between women and men, which have been shown to correlate with survival [21, 22]. Taken together, these data suggest an inherent difference in tumor biology between men and women in this subset of cancers. These differences may potentially influence not just the distribution, incidence, and behavior of these tumors, but may potentially be used to predict treatment outcomes and survival.

The aim of this study is to determine whether a difference in survival exists between men and women undergoing HIPEC and CRS for PC of appendiceal origin. To increase power over previous single-institution studies, we used the National Cancer Database (NCDB), a large, multi-institutional database, and multivariable regression analysis to control for potentially confounding variables. Furthermore, this study focused on patient sex as the principle factor. We hypothesized that women would have an independent survival advantage over men when undergoing HIPEC and CRS for PC from appendiceal origin.

## Methods

### Database

The NCDB is a joint project of the Commission on Cancer (CoC) of the American College of Surgeons (ACS) and the American Cancer Society. Data is sourced from over 1,500 CoC accredited facilities, accounting for more than 70% of newly diagnosed cancer cases nationwide. Data are available through organ specific Public User Files (PUF) that are HIPAA-compliant and de-identified. The ACS and the CoC have not verified and are not responsible for the analytic or statistical methodology employed, or the conclusions drawn from these data by the investigators. The NCDB PUFs used for this project spanned the years 2004–2014 and initially included colon, rectosigmoid, and peritoneum. An “exempt” status from the Institutional Review Board of the University of Oklahoma Health Sciences Center was granted for this project.

### Patient selection

The NCDB PUFs for the years 2004–2014 were reviewed for all patients that underwent HIPEC and cytoreductive procedures for cancers of appendiceal origin. The colonorgan-specific NCDB PUFs were used. Using the International Classification of Diseases for Oncology, Third Edition, we selected for patients with the appropriate cancer diagnosis under the subheading of Colon (C18), further specifying only of the appendix (C18.1). Then, we isolated patients who underwent chemotherapy and surgery simultaneously using “Systemic Surgery Sequence” (*RX_SUMM_SYSTEMIC_SUR_SEQ*) codes 5 and 6.

**Fig 1:**
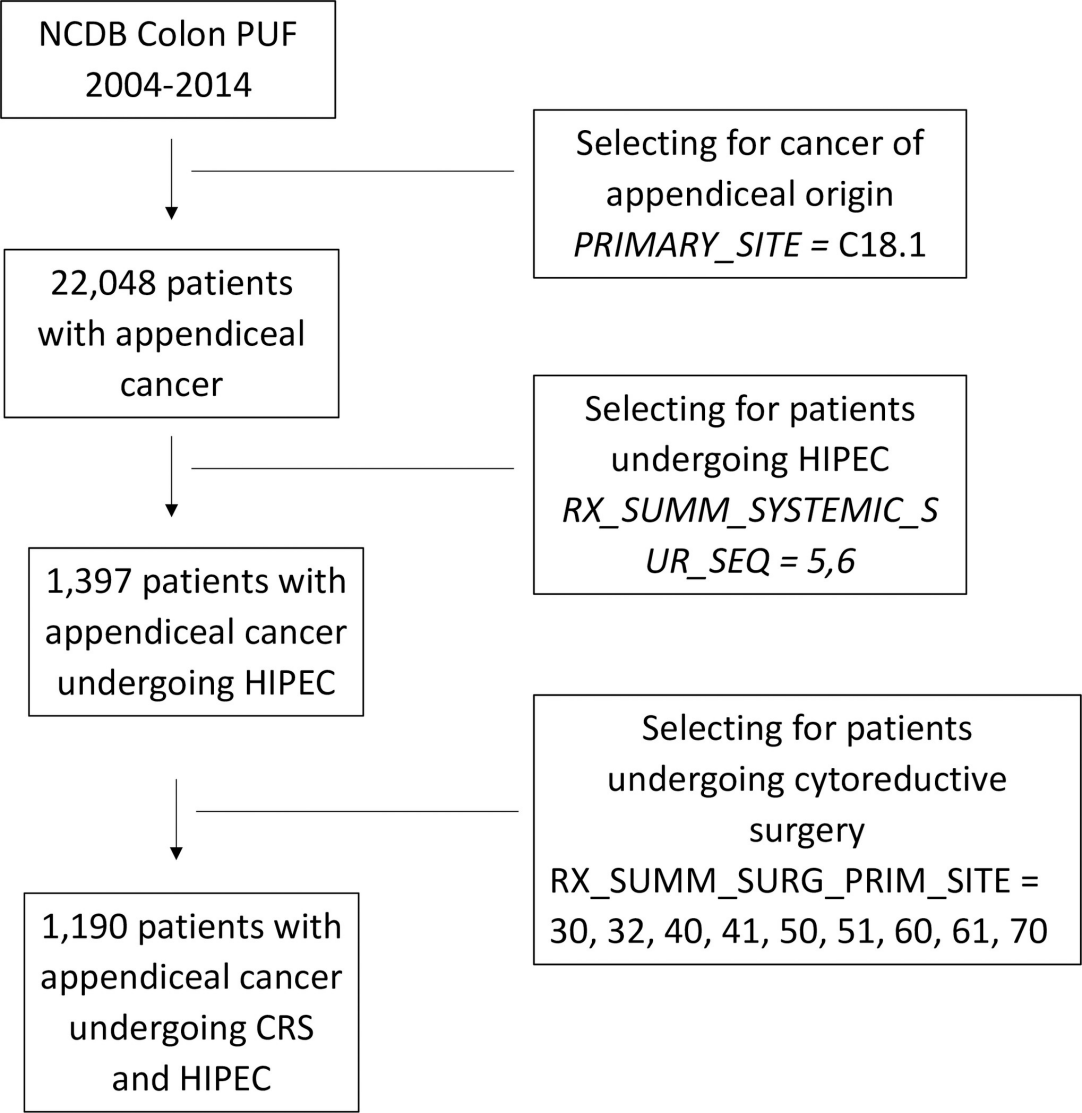
Selection of patients with appendiceal cancer undergoing HIPEC and CRS.

Next, we selected patients who underwent the appropriate cytoreductive procedures. We used “Surgical Procedure of the Primary Site” (RX_SUMM_SURG_PRIM_SITE) Site-Specific Surgery Codes for colectomy (30, 32, 40, 41, 50, 51) and proctocolectomy (60, 61, 70), including partial or complete resection of contiguous organs. A visual representation of our patient selection is depicted in Fig 1.

### Variables and outcomes

The demographic information, clinical variables, and outcomes analyzed were provided by the database. We evaluated variables including patient age, tumor histology, grade, and size, number of regional lymph nodes examined, hospital LOS, race, insurance status, median household income, education level, urban/rural environment, Charlson-Deyo score, surgical margins, 30-day re-admittance rate, and 30 and 90-day mortality. We used “Last Contact or Death” to estimate overall survival. Data were grouped and analyzed by sex.

The NCDB divides patient residence into 9 different classification codes based on population size and proximity to a metropolitan area. To simplify this classification scheme, we grouped the more densely populated counties (codes 1–3) together as “urban” and compared them to the less populated counties (codes 4–9) grouped together as “rural”. The NCDB has 31 categories of racial and ethnic groups. However, given the low number of patients representing many of the less populated ethnicities in our study, this classification scheme was simplified to white (code 01), black (02), and other (codes 4–98). The NCDB classifies insurance status into 6 categories, with 0 being uninsured, 1 being privately insured, 2–3 representing Medicaid and Medicare, respectively, 4 being “other government” insurance, and 9 representing an unknown insurance status. To simplify this on multivariable analysis, we grouped 0, 2–4 as “assisted insurance” and 1 as “privately insured”, comparing them directly to each other. Patients with unknown insurance status (9) were excluded from this analysis. Margin status was defined using the NCDB classification where 0: all margins grossly and microscopically negative—represented an R0 resection; 2: microscopic residual tumor—represented an R1 margin; and 3: macroscopic residual tumor- represented an R2 resection margin. R0 and R1 were combined to represent “complete cytoreduction”, previously defined as removal of all visible intraperitoneal and pelvic disease [23]. This was compared to R2, which represented an incomplete cytoreduction. Patients with margins that were not evaluated, not specified, or unknown were excluded from the margin analysis.

The NCDB measures education level using data from the 2012 American Community Survey that estimates the number of adults in a patient’s zip code that did not graduate from high school, and stratifies it based on percentages: 1: ≥21%, 2: 13%-20.9%, 3: 7%-12.9%, and 4: <7%. To simplify this stratification, we combined codes 1 and 2 to create a “Low graduation” group, where ≥13% of the population did not graduate high school. Codes 3 and 4 were then combined to make a “High graduation” group, in which <13% of the population did not graduate from high school.

### Statistical analysis

All data was extracted from the NCDB database and imported into SAS (version 9.4 SAS Institute, Cary, NC) for statistical analysis. Unadjusted differences between the two sexes were evaluated using chi-square test or Fisher’s exact test for categorical variables and Student’s t-test was used for continuous variables. The associations between the outcome variable and sex were further analyzed by using multivariable logistic regression analysis accounting for potential confounders. Kaplan-Meier curves were used to estimate survival with differences in survival between women and men compared with the log-rank test. Statistical significance was determined based on an alpha of 0.05, and variables that were statistically significant on univariate analysis were included in a multivariable Cox proportional hazards model., Results are reported in hazard ratios (HR) with 95% confidence intervals (95% CI).

## Results

A total of 1,190 patients with PC from appendiceal origin who underwent HIPEC and CRS were analyzed. Demographic data is presented for patients in Table 1. There were no significant differences between men and women in mean age, insurance status, education level, median household income, urban versus rural living area, or Charlson-Deyo scores. However, more African American women were represented in our patient population compared to African American men, 60 (5.12%) versus 24 (2.05%), p = 0.0096.

**Table 1:** Demographics and pre-existing comorbidities of patients undergoing HIPEC and CRS by gender.

Factor	Women		Men		
	(n = 611)		(n = 579)		
	Count	Percent	Count	Percent	P-value
**Home Address**					
Metro	505	85.7%	462	84%	0.4129
Urban/Rural	84	14.3%	88	16%	
**Age, Mean (STD)**					
	54.2	11.8%	54.4	12.4%	0.7245
**Race**					
White	524	85.8%	522	90.1%	0.0005
Black or African American	60	9.8%	24	4.2%	.
Other	27	4.4%	33	5.7%	.
**Insurance**					
Uninsured	10	1.7%	14	2.4%	0.5301
Private	441	72.8%	407	70.7	.
Government	155	25.6%	155	26.9%	.
**Charlson-Deyo**					
0	526	86.1%	513	88.6%	0.4117
1	72	11.8%	57	9.8%	.
≥2	13	2.1%	9	1.6%	.
**High School Diploma**					
≥13% non-graduates	201	33.4%	189	33.0%	0.883
<13% non-graduates	401	66.6%	384	67.0%	.

There were no differences between sexes in tumor grade, TNM stage, size, number of nodes examined, or hospital readmission. Oncologic and surgical outcomes data are represented in Table 2. While histology has been shown to affect survival in patients with PC from AMN, there were no significant differences in histology between sexes in the patient cohort. A breakdown of patient tumor histology is shown in Table 3. On univariate analysis, men had significantly longer median LOS than women, 17.1 versus 14.6, p = 0.0499. Additionally, women had fewer diagnostic biopsies performed than men, (6.1% vs 16.4%) and more women had no diagnostic procedures performed prior to resection compared to men (74.2% vs 53.4%, p<0.0001). There was no difference in the rates of R0/R1 versus R2 resection between men and women, with 73.2% of men vs 70.2% of women having R0/R1 resections documented, and 10.5% of men vs 7.9% of women having R2 resections (p = 0.2186). More women received single agent chemotherapy compared to men (65.3% vs 59.1%, respectively), while more men had multi-agent chemotherapeutic regimens (38.2% vs 30.9%, respectively, p = 0.0262). Interestingly, while 30-day mortality was low, it was higher for men compared to women, 8 (1.7%) vs 2 (0.4%), p = 0.0589, and 90 day mortality was significantly worse for men, 22 (4.6%) vs 10 (2.0%), p = 0.0295.

**Table 2:** Outcomes of patients undergoing HIPEC and CRS by gender-NCDB.

Factor	Women		Men		
	(n = 611)		(n = 579)		
	Count	Percent	Count	Percent	P-value
**Grade, n (%)**					
Well	246	40.3%	203	35.1%	0.2527
Moderate	130	21.3%	143	24.7%	
Poor	86	14.1%	98	16.9%	
Undifferentiated	13	2.1%	12	2.1%	
Unknown	136	22.3%	123	21.2%	
**Surgical Margins, n (%)**					
R0/R1	429	70.2%	424	73.2%	0.2186
R2	48	7.9%	61	10.5%	
**Chemotherapy, n (%)**					
Single-agent	399	65.3%	342	59.1%	0.0262
Multi-agent	189	30.9%	221	38.2%	
Unknown	23	3.8%	16	2.8%	
Mean # of Positive Lymph nodes (SD)**					
	4.7	4.3%	4.9	7.5%	0.8454†
**Readmission**					
	43	7.0%	47	8.1%	0.7272
**Reason for Readmission n (%)**					
Unplanned	33	5.4%	42	7.3%	0.3003
Planned	9	1.5%	3	0.5%	
Both	1	0.2%	2	0.4%	
Unknown	25	4.1%	21	3.6%	
**30-day Mortality, n (%)**					
	2	0.4%	8	1.7%	0.0589
**90-day Mortality, n (%)**					
	10	2.0%	22	4.6%	0.0295
**Mean Hospital Length of Stay (SD)**					
	14.6	19.7%	17.2	20.4%	0.0499†

† = p-value for Students T-test

# = number, NOS = not otherwise specified

**Only patients with positive nodes

**Table 3:** Tumor histology of patients undergoing HIPEC and CRS by gender.

Histology	Women		Men		
	(n = 598)		(n = 572)		
	Count	Percent	Count	Percent	P-value
**Adenocarcinoma**	56	9.36%	64	11.19%	0.66*
**Carcinoid**	23	3.85%	21	3.67%	
**Goblet Cell**	18	3.01%	19	3.32%	
**Mucinous**	456	76.25%	435	76.05%	
**Signet ring cell**	45	7.53%	33	5.77%	

*Chi-Square, Frequency Missing = 20.

**Fig 2:**
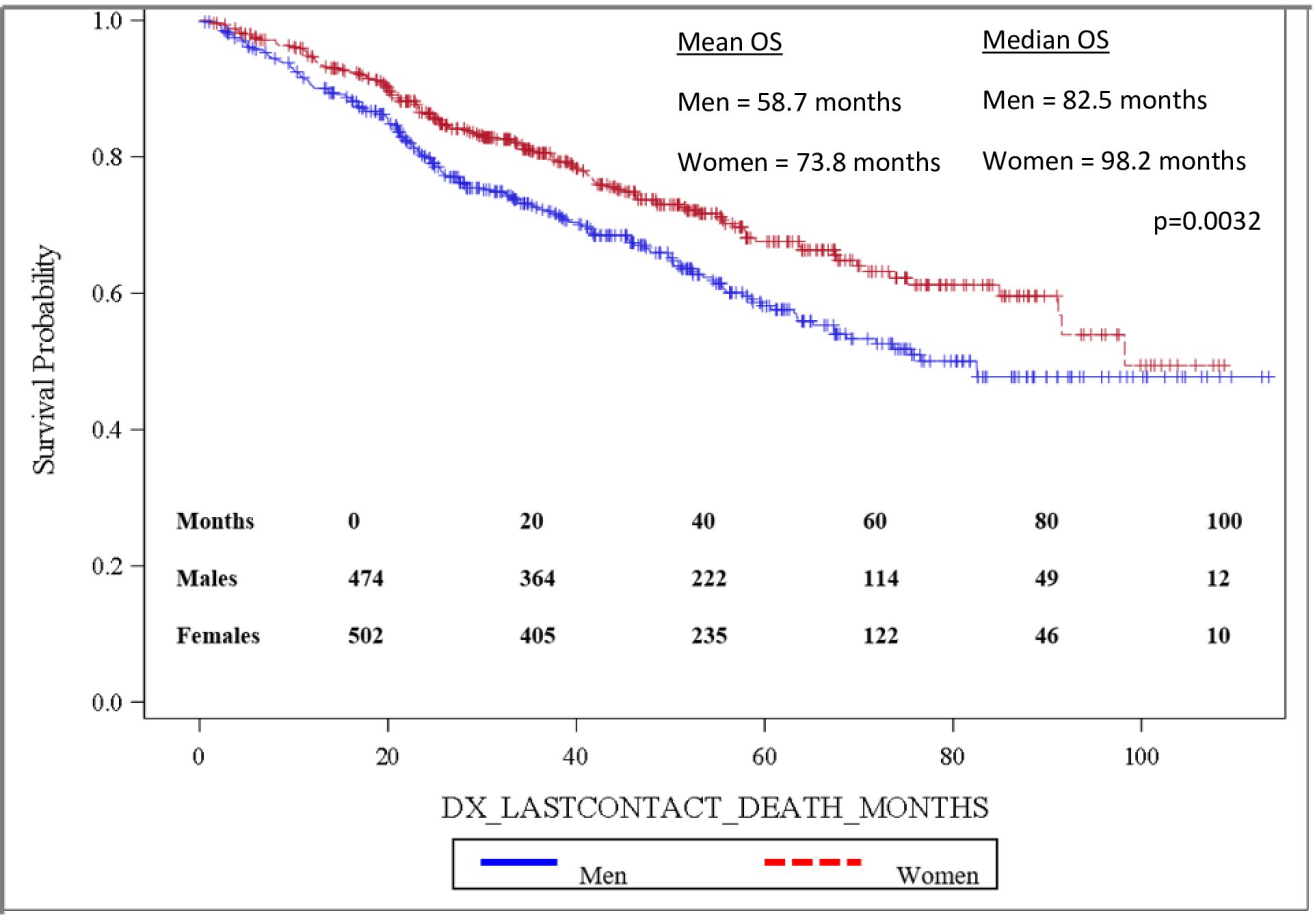
Overall survival by patient sex.

**Fig 3:**
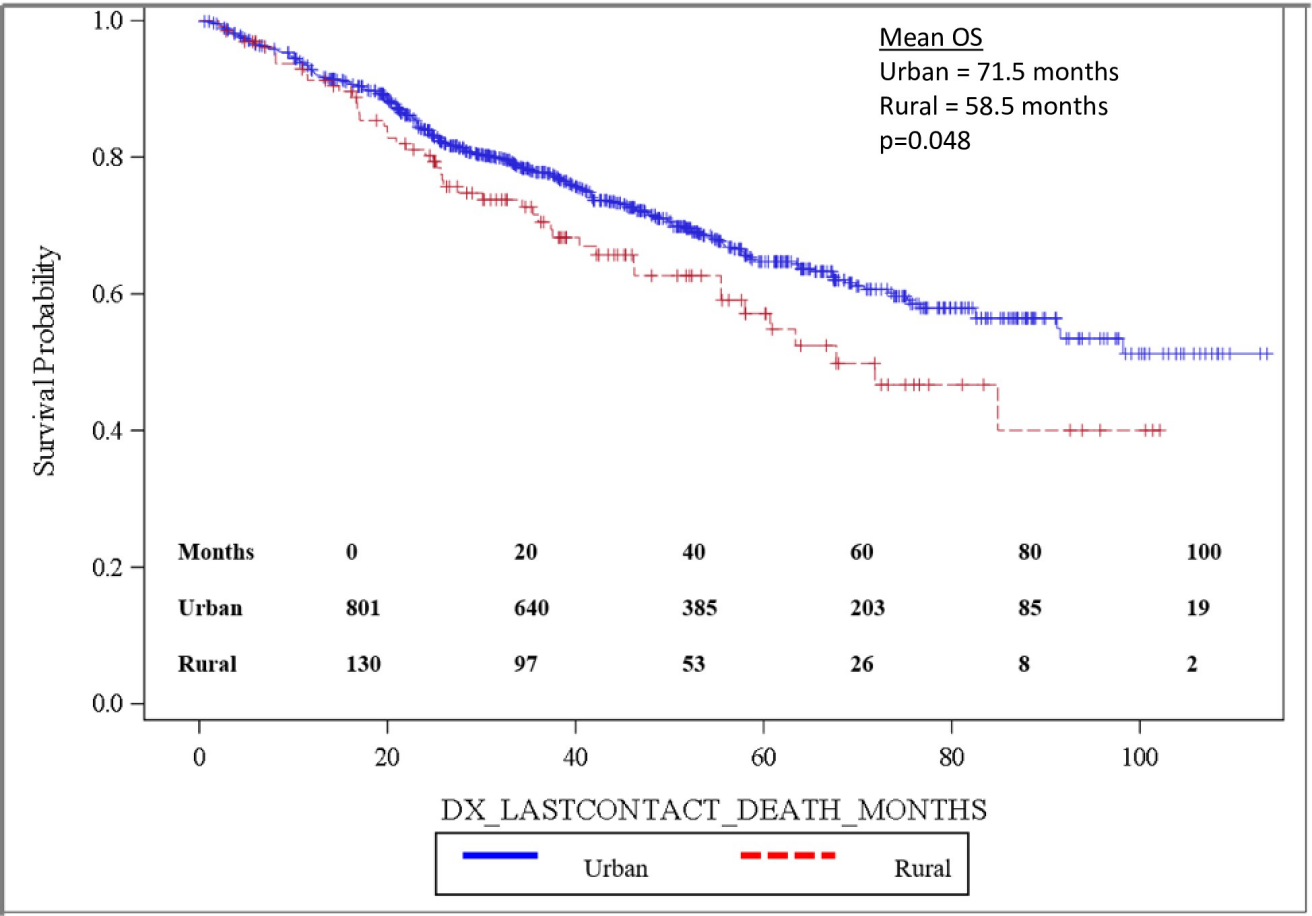
Overall survival by residential environment.

**Fig 4:**
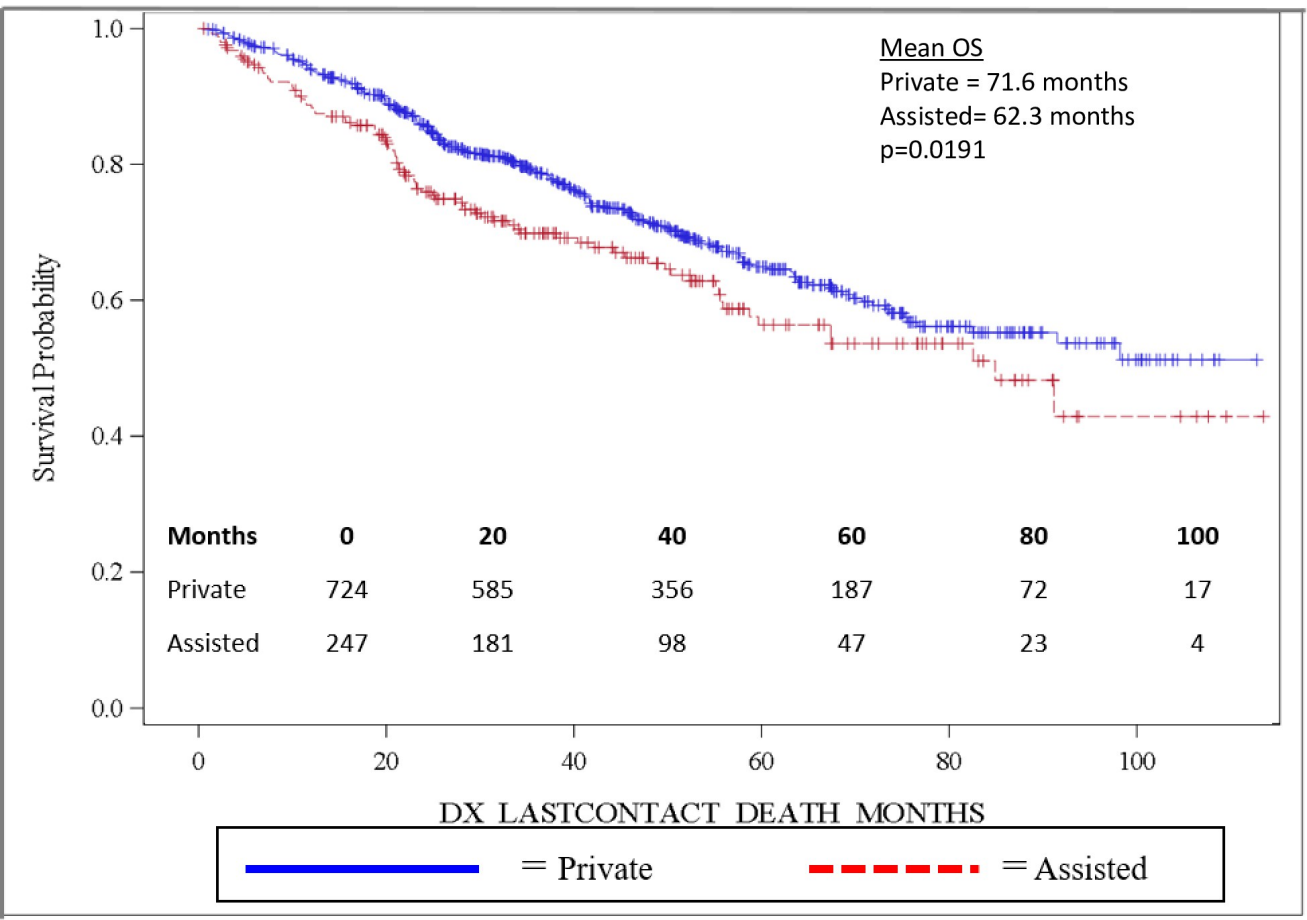
Overall survival by insurance status.

**Fig 5:**
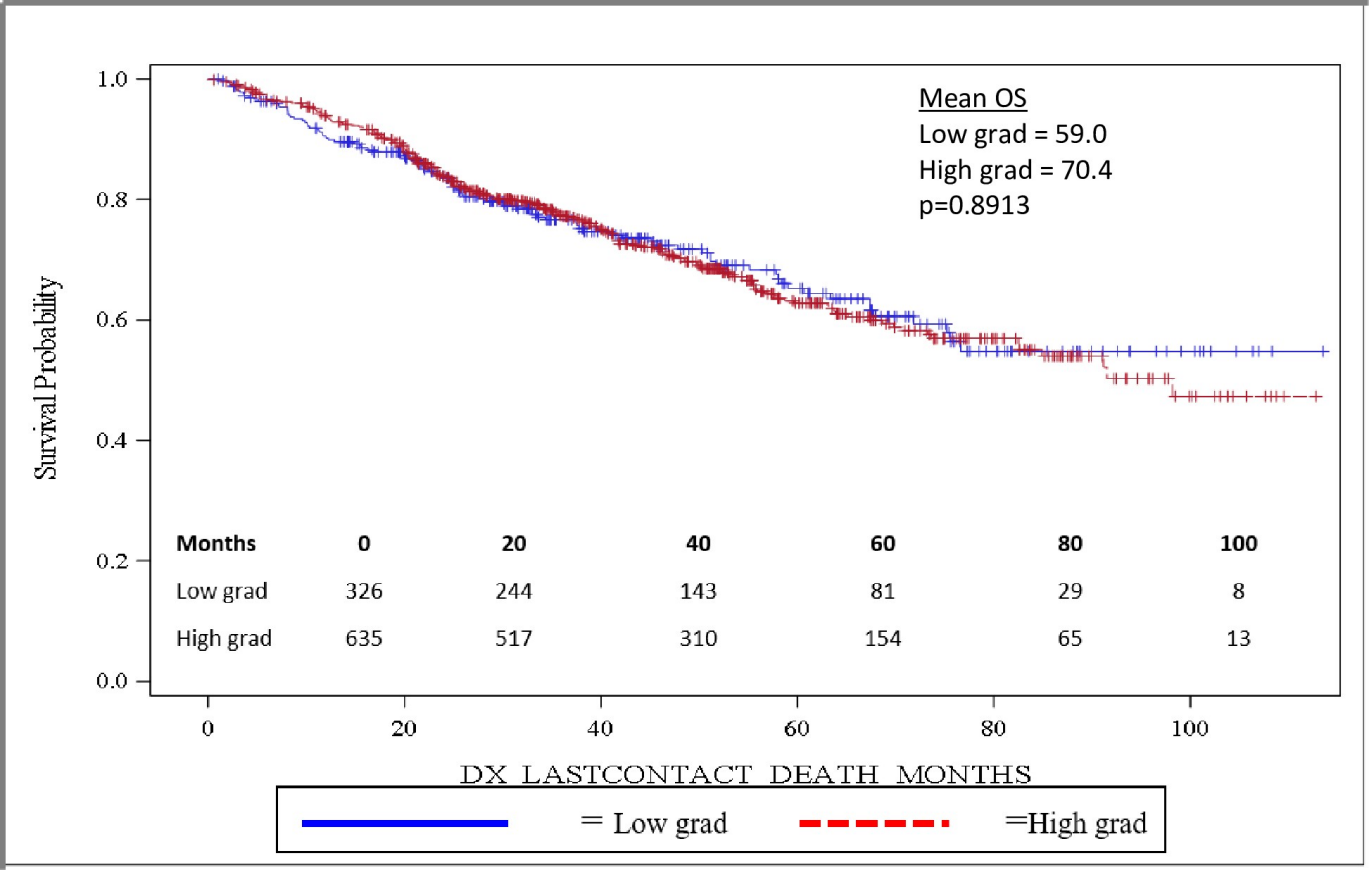
Overall survival based on high school education.

Importantly, OS was significantly longer for women than for men, with mean and median OS 73.8 months and 98.2 months for women and 58.7 months and 82.5 months for men, respectively (p = 0.0032). OS is depicted in the Kaplan-Meier curve in Fig 2. On multivariable analysis, male sex (HR: 1.444, 95% CI: 1.141–1.827, p = 0.0022) was found to independently predict worse OS. In addition, increasing age (HR: 1.017, 95% CI: 1.006–1.027, p = 0.0017) was also found to be an independent risk factor for worse OS. When grouped based on urban versus rural environment, patients living in an urban environment had a significant survival advantage over those living in a rural environment, with urban patients having a mean OS of 71.5 months versus 58.5 months for rural patients, p = 0.048 (Fig 3). When OS was analyzed based on insurance status, patients who were privately insured had a significant survival advantage over those who were uninsured or had government-based insurance, with privately insured patients having a mean OS of 71.6 months versus 62.3 months for patients with “assisted insurance” (p = 0.0191) (Fig 4). On multivariable analysis, insurance status was not found to be independently predictive of survival (HR = 0.761, 95% CI: 0.556–1.042, p = 0.0882). When grouped based on education level in Fig 5, another marker of socioeconomic status, there was no difference in mean OS between patients living in areas with low rates of high school graduation, 59.0 months, versus high rates of high school graduation, 70.4 months (p = 0.8913). Additionally, when OS was analyzed based on race, no difference was observed.

## Discussion

The objective of this study was to determine whether sex impacts survival of patients with PC from stage IV cancer of appendiceal origin undergoing treatment with HIPEC and CRS. This study found that male sex is an independent predictor of worse OS compared to female sex. Furthermore, we found that increasing age was an independent predictor of worse OS. Finally, we found that living in a metropolitan area was associated with improved OS on univariate analysis but was not an independent predictor of improved OS following treatment for PC for AMN on multivariable analysis.

Treatment and prognosis of PMP for AMN has changed significantly since improved survival has been found with HIPEC and CRS compared to previously employed therapies, including chemotherapy, repeated peritoneal draining and surgical debulking in some retrospective series [10, 11, 24]. Since this shift in practice patterns, multiple factors have been examined with regard to their effect on outcomes following HIPEC and CRS, including: age, performance status, histopathologic subtype, completeness of cytoreduction, lymph node status, pre-operative chemotherapy, tumor markers, and PCI [13, 15, 25–27]. Interestingly, common perioperative clinical factors affecting outcome, including postoperative surgical complications and extent of CRS, have not been shown to consistently affect survival for patients undergoing HIPEC and CRS for cancers of appendiceal origin [12, 14, 16, 28]. However, our study showed an increased 30 and 90-day mortality for men compared to women, suggesting that perioperative morbidity affects some element of survival for these patients. The limited reporting of postoperative complications in the NCDB remains a notable limitation. However, given that multiple other studies have failed to show a significant correlation between perioperative morbidity and long-term survival in this specific patient population, postoperative complications are likely not the driving force behind the significant survival advantage for women noted in this study.

Patient sex is a common factor included in descriptive analyses of studies investigating malignancies of colorectal origin, including cancer of the appendix. However, to our knowledge, the impact of sex on survival in appendiceal malignancies has not studied using a multi-institutional, national database in a multivariable analysis with patient sex as the primary outcome of interest. Several previous single-institution studies found no significant correlation between sex and survival [13–15]. Other studies found a survival advantage for women compared to men; however, these studies did not explore patient sex as a specific variable of interest [10, 16, 29]. Ung et al examined the impact that sex has on survival in a retrospective, single-institution study, where they found that median progression free survival (PFS) for women was 50.7 months compared to 31.5 months for men (p = 0.07), and multivariable analysis demonstrated an independent survival advantage for women over men (HR: 3.03, 95% CI: 1.39–6.60, p = 0.005) [17]. Using a multi-institutional, national database, this study supports the finding that male sex is an independent risk factor for worse OS compared to women. To our knowledge, this study represents the largest, multi-institutional cohort of patients with appendiceal neoplasms undergoing treatment with HIPEC and CRS demonstrating a survival advantage for women as compared to men in a multivariable regression analysis. Validating the findings from previous studies with a higher number of patients spanning multiple institutions strengthens the assertion that patient sex is an important prognostic factor in this patient population.

While the findings of our study and those of previous studies demonstrate a survival advantage for women, the underlying biology responsible for this clinical observation remains unclear. In patients with CRC, several studies have demonstrated a survival advantage for women over men [19, 30, 31]. While early identification and the management of comorbid conditions may influence these findings [30], this survival advantage has been shown even when women receive less aggressive chemotherapeutic regimens [19]. While these data cannot be directly applied to tumors of the appendiceal epithelium, it is possible there is overlap. Ultimately, the mechanisms underlying this relationship remain unclear.

It is well-established that pseudomyxoma peritonei from AMN is a heterogenous disease including multiple different histopathologic subtypes [1, 10, 14, 26]. Furthermore, these subtypes have been shown to significantly affect survival in patients with AMN [13, 14, 26]. Interestingly, multiple different systems exist for classifying this diverse group of tumors, with most basing their groupings on differences in survival. These tumors are challenging to stage, which is further complicated by the multiple different classification systems. Ronnett et al classified patients into 3 groups: disseminated peritoneal adenomucinosis (DPAM), peritoneal mucinous carcinomatosis (PMCA), and PMCA-I, including PMCA with intermediate or well-differentiated features [32]. The authors demonstrated a difference in survival, with DPAM surviving longer than PMCA-I, which in turn had improved survival over PMCA. The NCDB uses International Classification of Diseases for Oncology, Third Edition, which classifies tumors of appendiceal origin broadly as malignant carcinoid tumors, goblet cell carcinoids, adenocarcinoma, mucinous adenocarcinoma, and signet ring cell adenocarcinoma. The distribution of histologies in our patient population is shown in Table 3. Importantly, there was no significant difference between men and women with regards to histology, suggesting that the survival advantage noted for women cannot be readily attributed to disparity in histopathologic subtype.

The impact of age at diagnosis on survival in patients with PMP from AMN has been previously examined by multiple studies with disparate findings. Several studies report no prognostic significance for age with regards to survival [14, 33, 34], while others show that older age is a risk factor for worse OS [10, 12, 16, 25]. The reasons why age may influence outcomes are likely multifactorial, including increased postoperative morbidity, decreased performance status, different age distributions for appendiceal malignancies, and potential lower doses of chemotherapeutic drugs and perfusion times [15, 25]. However, a study by Votanopoulos et al examining outcomes in elderly patients >70 years old undergoing HIPEC and CRS for a variety of GI malignancies, including appendiceal neoplasms, concluded that age alone was not a contraindication to undergoing treatment [35]. The authors advocate for focusing on the type of primary, performance and nutritional status, and the ability to perform complete cytoreduction to assess operative candidacy, as opposed to chronologic age. Conversely, younger age can often be a negative prognostic factor when dealing with malignant disease, including unique socioeconomic challenges and distinct, often more aggressive tumor biology. However, Dhir et al suggest that younger aged patients with GI malignancies benefit similarly from HIPEC/CRS when compared to middle-aged patients [36]. Thus, while our study found advancing age as an independent predictor of worse OS, it should not be evaluated in isolation but rather acknowledged as a known risk factor and included in pre-operative risk assessment.

Our study has several limitations. These data were derived from a national, multi-institutional database, which has the advantage of providing a large, diverse patient population. However, the NCDB, like many large databases, is limited in the granularity and quality of data, susceptible to bias and type I statistical error [37]. For instance, previous studies have demonstrated that a higher PCI is a negative prognostic factor that affects OS in this patient population [9, 10]. The NCDB does not provide a PCI score or data with which to calculate PCI, thus we were unable to control for tumor burden and extent of disease using this accepted predictive scoring system. Similarly, completeness of cytoreduction (CC) has been shown to be an important predictor of improved survival in patients with PC from GI malignancies, including those arising from the appendix [9, 10, 23]. CC 0–1 indicates a complete cytoreduction, with removal of all visible disease or residual nodules <2.5mm, and CC 2–3 represents an incomplete cytoreduction, with residual disease ≥2.5mm in size [38]. We are unable to account for this prognostic factor using the NCDB. However, we estimated completeness of cytoreduction by using NCDB margin status, combining R0/R1 to represent a complete cytoreduction (CC 0–1), and R2 to represent an incomplete cytoreduction (CC 2–3). The rate of complete cytoreduction varies throughout the literature, ranging from 65% - 89% [9–12, 23]. Our rate of complete cytoreduction was 70.2% in women, 73.2% in men, similar to rates quoted in the literature; importantly, there was no significant difference in rate of completeness of cytoreduction between men and women. Furthermore, this heterogeneous group of malignancies is challenging to classify, complicated further by a lack of standardized histopathologic reporting system, as previously mentioned. Recent attempts to standardize the process may mitigate this limitation for future studies but is not applicable to our study period [39]. Therefore, we acknowledge that a small percentage of the patients in our study may have been misdiagnosed at their initial institution, mislabeled in the dataset, or inaccurately included or missed by the patient selection criteria.

While the NCDB has minimal data regarding postoperative complications, it does have the capacity to estimate long-term survival as it tracks the date of last contact or death in months from the date of diagnosis. Additionally, while the NCDB does provide information regarding chemotherapeutic regimens, including timing of treatment and the number of agents used, it lacks drug-specific data. However, there is no universal agreement on which chemotherapy regimen is superior for this disease. In fact, previous studies suggest no statistically significant survival difference between the most commonly used drugs, mitomycin C and oxaliplatin [40]. It should also be noted that these data, gathered from the robust NCDB dataset, span the years 2004–2014. Several years have passed since these data were available, and advances in chemotherapy have been made, including notable developments in combination chemotherapeutic regimens, targeted biologic agents and immunotherapy. However, these newer agents have not been studied as treatment in this disease process except in patients for whom surgery is not an option due to advanced, unresectable disease or severe medical co-morbidities, and therefore should have limited relevance to this patient population [41, 42]. Furthermore, as mentioned previously, HIPEC for appendiceal cancer is most frequently performed with mitomycin C and oxaliplatin, with cisplatin and 5-fluorouracil being other commonly used agents [1]. All of these cytotoxic drugs were in use and available during our study period. Lastly, while our findings clearly demonstrate a survival advantage for women over men, it provides little insight into the mechanism underlying this finding. However, the strength of large, multi-institutional databases is their effectiveness at uncovering clinical associations and relevant outcomes that are not easily noticed in smaller, more granular databases.

## Conclusion

Among patients with PMP from AMN undergoing treatment with HIPEC and CRS, male sex is an independent risk factor for worse OS. There is no evidence that this survival advantage was attributable to age at diagnosis, histopathologic subtype, insurance status, race, patient living environment or Charlson-Deyo score. Additionally, increasing age was an independent predictor of worse OS, while living in a metropolitan area was associated with improved OS on univariate analysis alone. While 30 and 90-day mortality were higher in men compared to women, the NCDB lacks data regarding postoperative morbidity and mortality, limiting our interpretation of these data. Further studies are needed to determine the mechanism underlying this notable survival advantage as it has the potential to affect clinical decision making, patient counseling, interpretation of previous and current studies, the design of future prospective trials, and may influence treatment modalities.
